# Smoking Cessation and Cardiovascular Disease Risk Factors: Results from the Third National Health and Nutrition Examination Survey

**DOI:** 10.1371/journal.pmed.0020160

**Published:** 2005-06-28

**Authors:** Arvind Bakhru, Thomas P. Erlinger

**Affiliations:** **1**University of Rochester Medical Center, School of MedicineRochester, New YorkUnited States of America; **2**Department of Epidemiology and Public Health, Yale University School of MedicineNew Haven, ConnecticutUnited States of America; **3**Department of Epidemiology, Johns Hopkins University Bloomberg School of Public HealthBaltimore, MarylandUnited States of America; **4**Department of Medicine, Johns Hopkins University School of MedicineBaltimore, MarylandUnited States of America; **5**Welch Center for Prevention, Epidemiologyand Clinical Research, Johns Hopkins Medical Institutions, Baltimore, MarylandUnited States of America; University of QueenslandAustralia

## Abstract

**Background:**

Cigarette smoking is a major risk factor for the development and progression of cardiovascular disease. While smoking is associated with increased levels of inflammatory markers and accelerated atherosclerosis, few studies have examined the impact of smoking cessation on levels of inflammatory markers. The degree and rate at which inflammation subsides after smoking cessation are uncertain. It also remains unclear as to whether traditional risk factors can adequately explain the observed decline in cardiovascular risk following smoking cessation.

**Methods and Findings:**

Using data from 15,489 individuals who participated in the Third National Health and Nutrition Examination Survey (NHANES III), we analyzed the association between smoking and smoking cessation on levels of inflammatory markers and traditional cardiovascular risk factors. In particular, we examined changes in C-reactive protein, white blood cell count, albumin, and fibrinogen. Inflammatory markers demonstrated a dose-dependent and temporal relationship to smoking and smoking cessation. Both inflammatory and traditional risk factors improved with decreased intensity of smoking. With increased time since smoking cessation, inflammatory markers resolved more slowly than traditional cardiovascular risk factors.

**Conclusion:**

Inflammatory markers may be more accurate indicators of atherosclerotic disease. Inflammatory markers returned to baseline levels 5 y after smoking cessation, consistent with the time frame associated with cardiovascular risk reduction observed in both the MONICA and Northwick Park Heart studies. Our results suggest that the inflammatory component of cardiovascular disease resulting from smoking is reversible with reduced tobacco exposure and smoking cessation.

## Abstract 2

Analyzing inflammatory markers quantifies the cardiovascular risk of smoking, and suggests that inflammation induced by smoking is reversible, with markers returning to baseline by 5 years after cessation.

## Introduction

In addition to the role of smoking in cancer initiation and promotion, cigarette smoking accelerates atherogenic cardiovascular disease in both a dose- and a duration-dependent manner through several concurrent pathways. Smoking incites an immunologic response to vascular injury, described as oxidative stress leading to lipid peroxidation, endothelial cell dysfunction, and foam cell proliferation in the tunica media [1,2]. Smoking also enhances platelet aggregation, impairs lipoprotein metabolism, depresses high-density lipoprotein (HDL) cholesterol, and reduces distensibility of vessel walls [3,4].

Cigarette smoking is associated with increased levels of inflammatory markers. During the acute phase of inflammatory states, there are quantifiable increases in C-reactive protein, white blood cell count, and fibrinogen, and decreases in serum albumin [5–8]. Acute inflammatory markers have been shown to be both prognostic and predictive of future cardiovascular events in several populations. For example, C-reactive protein has been discussed extensively as a marker of cardiovascular disease risk [9–16].

C-reactive protein in particular is also increasingly being implicated in the pathogenesis of atherosclerosis [17–19]. C-reactive protein is a pentaxin, highly conserved across species [17,20,21] and stimulated synergistically by both IL-6 and IL-1β [22–25]. Previously believed to be synthesized by the liver, recent evidence suggests that C-reactive protein is also produced at the site of atherosclerosis by smooth muscle cells [17]. In the vessel wall, it induces expression of adhesion molecules on endothelial cells and increases monocyte chemotactic protein-1, which attracts monocytes and T cells into the vessel wall [26–28]. Additionally, C-reactive protein reduces endothelial nitric oxide synthase, upregulates the proatherosclerotic NF-κB pathway, enhances low-density lipoprotein (LDL) uptake by macrophages, which become foam cells, and facilitates T-cell- and complement-mediated destruction and apoptosis of the endothelium [2,26,29–31]. Thus, C-reactive protein is increasingly described not only as a marker of the inflammatory response, but also as a mediator in the pathogenesis of atherosclerotic cardiovascular disease [2,27]. Other inflammatory markers may also have causal properties, but are not as well understood. The inflammatory response therefore not only indicates atherosclerotic potential, but may accelerate atherosclerosis.

Several studies have described a prevalent inflammatory state in smokers. Despite the known impact of smoking on cardiovascular disease progression, few studies have examined the impact of smoking cessation on levels of inflammatory markers or on cardiovascular risk reduction [6,32]. The level to which the inflammatory response subsides following smoking cessation and the rate at which the inflammatory response subsides are uncertain. Furthermore, it remains unclear whether traditional risk factors can adequately explain the decline in cardiovascular risk following smoking cessation.

Using the Third National Health and Nutrition Examination Survey (NHANES III), a population-based representative sample of United States adults, we investigated the association between smoking and smoking cessation and levels of inflammatory markers and traditional cardiovascular risk factors. We examined the association between changes in the inflammatory markers—C-reactive protein, white blood cell count, albumin, and fibrinogen—and the traditional risk factors—total cholesterol, HDL cholesterol, triglycerides, systolic blood pressure, and diabetes—with decreased smoking intensity and increased time since smoking cessation. Our primary objective was to investigate changes in C-reactive protein in an effort to characterize the excess cardiovascular risk associated with smoking and any associated decline in risk with smoking cessation. We also aimed to characterize whether the inflammatory markers or traditional risk factors explained observed cardiac risk reduction following smoking cessation.

## Methods

### Study Population

The NHANES III survey and data collection procedures have been described in detail elsewhere [33,34]. Briefly, NHANES III was a national probability survey conducted between 1988 and 1994. This survey used a complex, multi-stage, stratified cluster sampling design to obtain a representative sample of the non-institutionalized civilian United States population. Participation included a home examination and a visit to a mobile examination center in one of 89 locations. For those unable to travel to a mobile examination center, a special home visit was arranged.

Of the 19,618 persons aged 18 and over included in the NHANES III population, we excluded 3,021 persons for whom C-reactive protein levels were missing, given that this was our primary marker of interest. Another 292 persons were excluded because they were pregnant, and 816 people were excluded because they reported cigar, pipe, snuff, or chewing tobacco use. Data for 15,489 persons were analyzed.

### Measures

Self-reported race/ethnicity was categorized as non-Hispanic white, non-Hispanic black, Mexican-American, or other. The self-reported poverty to income ratio, a marker of socioeconomic status used in multiple prior studies [35,36], was classified as <1.5, 1.5–3, or >3.

Self-reported clinical factors were dichotomized for analysis. Participants reported on use of non-steroidal anti-inflammatory drugs, estrogen replacement therapy, and vitamin supplements. Use of any alcohol in the past 24 h was measured by dietary recall questionnaire. Prior physician-diagnosed angina, myocardial infarction, or stroke was defined as prevalent atherosclerotic cardiovascular disease (ASCVD). Prevalent inflammatory disease was defined as the presence of rheumatoid arthritis, asthma, emphysema, or chronic bronchitis. Presence of acute illness was indicated by a positive answer to the question: “In the past few days have you had a cough, cold, or other acute illness?”

Additional clinical factors were measured by a combination of participant self-report and clinical exam and were also dichotomized for analysis. Hypertension was indicated by use of anti-hypertensive medication, an average systolic blood pressure over 140 mm Hg, or an average diastolic blood pressure over 90 mm Hg, over four readings. Diabetes mellitus was considered present if the participant reported physician diagnosis of diabetes mellitus (excluding diagnosis during pregnancy), was taking diabetes medications, had fasting plasma glucose over 7 mmol/l (126 mg/dl), or had non-fasting glucose levels over 11.1 mmol/l (200 mg/dl). Finally, serum cholesterol and triglyceride levels were measured enzymatically (Hitachi 704 analyzer, Boehringer Mannheim, Mannheim, Germany). LDL was calculated using the Friedewald equation and measured fasting triglycerides, total cholesterol, and HDL cholesterol. LDL cholesterol and triglycerides were included as continuous variables and were logarithmically transformed to approximate the normal curve.

Smoking history was categorized based on both self-report and serum cotinine levels. Persons who gave a history of current smoking and/or had serum cotinine levels greater than 56.8 nmol/l (10 ng/ml) were considered current smokers. Current smokers were categorized into four roughly equal groups, based on number of cigarettes per day: 1–9, 10–19, 20–29, and over 30 cigarettes per day. Former smokers were defined by self-report as having smoked over 100 cigarettes in their lifetime and as having quit smoking. Former smokers were categorized by years since smoking cessation. Since the zero year since cessation point is subject to misclassification bias, the following levels were used in analyses: <1, 1–3, 3–5, 5–7, 7–9, and >9 ysince cessation. Never smokers were defined by self-report and as having serum cotinine levels under 56.8 nmol/l (<10 ng/ml). Passive smokers with cotinine levels over 56.8 nmol/l (10 ng/ml) were categorized as smokers at the lowest dose-intensity level.

Our main outcome variable, C-reactive protein, was measured using a latex-enhanced Behring Nephelometer Analyzer System (Dade Behring, Deerfield, Illinois, United States). Because the presence or absence, rather than the absolute level, of C-reactive protein appears to be associated with cardiovascular risk, we compared undetectable versus detectable levels of this marker. This is consistent with previous research, given the nonlinearity of C-reactive protein interpretation [37,38]. Since the limit of detection for the latex-enhanced nephelometry assay was 2.1 mg/l, we defined undetectable as less than 2.1 mg/l. Other outcome variables were treated continuously. White blood cell count was determined using a fully automated Coulter S-PLUS JR hematology analyzer (Beckman Coulter, Fullerton, California, United States). Albumin was measured using the bromocresol purple method. Fibrinogen was measured using a quantitative assay of clotting time compared to a known standard.

### Statistical Methods

The distributions of traditional risk factors, inflammatory markers, and clinical and sociodemographic factors were examined by smoking status. Chi-square and analysis of variance tests, as appropriate, were performed to test for statistically significant differences. Variables identified as potential confounders by these bivariate analyses were included in multivariate models, as were the cofactors believed to be clinically relevant.

For multivariate analyses, our primary independent variables were smoking intensity and time since smoking cessation. Our primary dependent variables were the inflammatory markers—C-reactive protein, fibrinogen, white blood cell count, and serum albumin—and the traditional risk factors—systolic blood pressure, total cholesterol, triglycerides, HDL, LDL, diabetes, and alcohol use. Two sets of multivariate models were developed. The first set of analyses (minimally adjusted) were adjusted for age, sex, and race, given previously reported differences in the distribution of inflammatory markers in those groups. The second set of analyses (fully adjusted) were adjusted for all covariates and potential confounders: age, sex, race, poverty to income ratio, body mass index, prevalent cardiovascular disease, prevalent diabetes, prevalent chronic inflammatory condition, current acute illness, and use of alcohol, non-steroidal anti-inflammatory medication, aspirin, and estrogen replacement.

For inflammatory markers and traditional risk factors that were measured continuously, linear regressions were used to model the relationship to smoking intensity and time since smoking cessation. Least square means were calculated. C-reactive protein (a dichotomous variable) was modeled with logistic regression, using never smokers as the reference category. Odds ratios, however, do not approximate risk ratios, given that the prevalence of elevated C-reactive protein is over 10%.

The dose–response relationship between both smoking intensity and time since smoking cessation and each of the outcomes was assessed using Mantel tests for trend. Three sets of tests were performed, evaluating the trends: (1) among current smokers by cigarettes per day; (2) among former smokers by time since cessation; and (3) among smokers, former smokers, and non-smokers, despite the potential nonlinearity between groups. To further explain the relationship between smoking exposure and C-reactive protein, to increase the power to detect a trend, and to reduce residual confounding, C-reactive protein was categorized into three levels: under 2.1 mg/l (undetectable), 2.1–9.9 mg/l (mildly elevated), and ≥10.0 mg/l (clinically significant).

Lastly, blocked group comparisons were done using adjusted chi-square tests. Inflammatory and traditional risk factors were compared across current, former, and never smokers to validate findings from previous studies.

All analyses were weighted to represent the total civilian, non-institutionalized United States population; they are, therefore, population prevalence estimates. Analyses were conducted in SAS (version 8.02; SAS Institute, Cary, North Carolina, United States) and SUDAAN (version 9.0; Research Triangle Institute, Research Triangle Park, North, Carolina, United States), incorporating sampling weights to account for nonresponse and oversampling. All analyses were conducted using two-sided tests with alpha set to 0.05.

## Results

Of the 15,489 persons in our sample, 7,665 were classified as never smokers, 3,459 were classified as former smokers, and 4,365 were classified as current smokers. The average time since smoking cessation for former smokers was 13 y. The average cotinine level for current smokers was 1,255 nmol/l (221 ng/ml); for both former and never smokers the average cotinine level was <3 nmol/l (<0.5 ng/ml). Weighted descriptive statistics are given in Table 1.

**Table 1:**
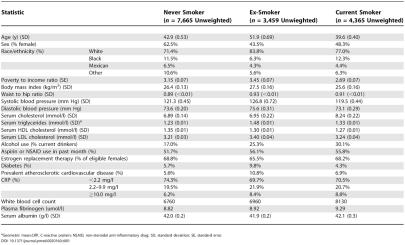
Descriptive Statistics of the NHANES III Sample Weighted to Reflect the United States Population

^a^Geometric mean.CRP, C-reactive protein; NSAID, non-steroidal anti-inflammatory drug; SD, standard deviation; SE, standard error.

Bivariate analyses of smoking status showed that among the inflammatory risk factors, unadjusted C-reactive protein, white blood cell count, and fibrinogen were all significantly and positively associated with smoking status (*p <* 0.01). Albumin levels were similar across smoking categories. Among traditional risk factors, smokers had higher total cholesterol and triglycerides, but were otherwise younger, had lower body mass index, and had lower blood pressure, and a smaller proportion were diabetic. Former smokers were most likely to have prevalent atherosclerotic cardiovascular disease.

Bivariate analyses of the inflammatory markers showed that C-reactive protein was significantly associated with older age (*p-*trend < 0.01 by 10-y age group), female sex (*p <* 0.01), and black race (*p <* 0.01). Serum albumin and serum fibrinogen showed similarly significant associations. White cell count was associated with race (*p <* 0.01), but not with age or sex. Among traditional risk factors, analyses showed that total cholesterol, triglycerides, HDL cholesterol, LDL cholesterol, and systolic blood pressure were each associated with age, sex, and race (all *p <* 0.01). Diabetes was associated with age and race only (*p <* 0.01). Alcohol use was associated with age and sex only (*p <* 0.01). All models, therefore, were adjusted for age, race, and sex.

**Figure 1:**
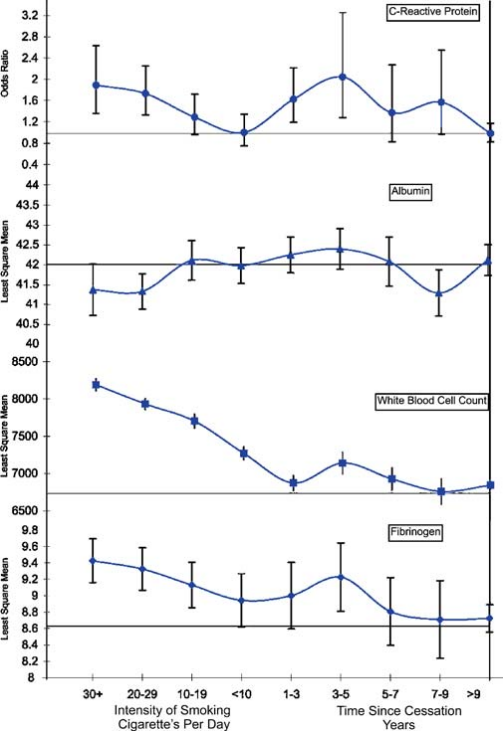
Trends in Inflammatory Markers with Smoking Intensity and Time since Cessation Are Given, Adjusted for Age, Sex, and Race

Table 2 displays abatement of the inflammatory response with (1) reduced intensity of smoking and (2) increased time since smoking cessation, adjusted for age, sex, and race. C-reactive protein, white blood cell count, and fibrinogen were all positively associated with increased smoking intensity (*p ≤* 0.01) and were all significantly negatively associated with time since smoking cessation (*p <* 0.05). Similarly, albumin showed a negative association with intensity of smoking (*p ≤* 0.01), but no relationship after cessation. All acute phase reactant inflammatory markers had significant trends overall, from current to former to never smokers (*p ≤* 0.01) (Figure 1).

**Table 2:**
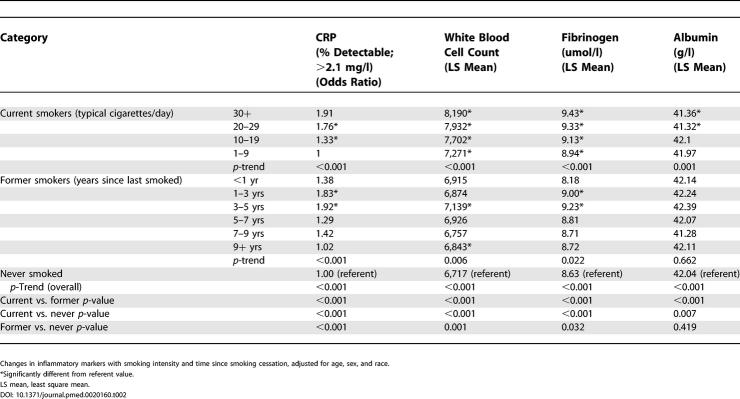
Levels of Inflammatory Markers by Smoking Status and Smoking Intensity

Changes in inflammatory markers with smoking intensity and time since smoking cessation, adjusted for age, sex, and race.

*Significantly different from referent value.

LS mean, least square mean.

**Figure 2:**
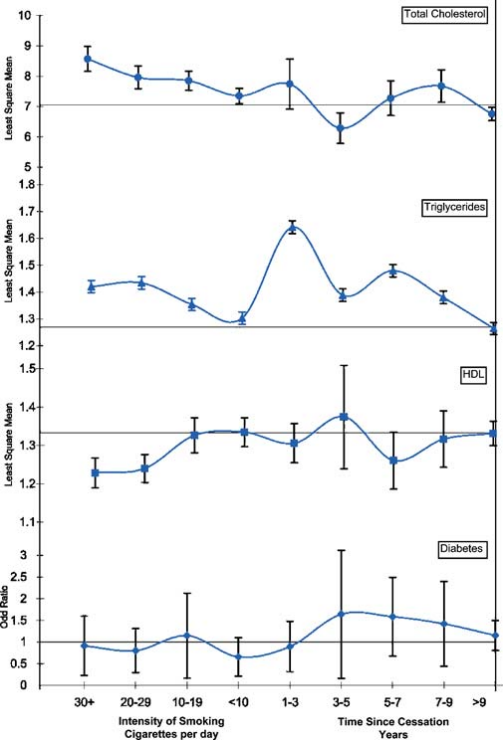
Trends in Traditional Risk Factors with Smoking Intensity and Time since Cessation Are Given, Adjusted for Age, Sex, and Race

Differences in the traditional cardiovascular risk factors are shown in Table 3, adjusted for age, sex, and race. Total and HDL cholesterol, triglycerides, alcohol usage, and systolic blood pressure all showed a dose-dependent association with smoking intensity (*p <* 0.05). Triglycerides and alcohol use showed time-dependent associations with smoking cessation (*p ≤* 0.01). Overall trends (from current to former to never smokers) were present for alcohol use, triglycerides, total cholesterol, and HDL cholesterol, as shown in Figure 2. In models fully adjusted for all covariates (not shown), dose-dependent associations persisted for HDL cholesterol and triglycerides (*p ≤* 0.01). Both total cholesterol and triglycerides showed an association with time since smoking cessation (*p =* 0.03 for both) and an overall trend from current to never smokers in fully adjusted models.

**Table 3:**
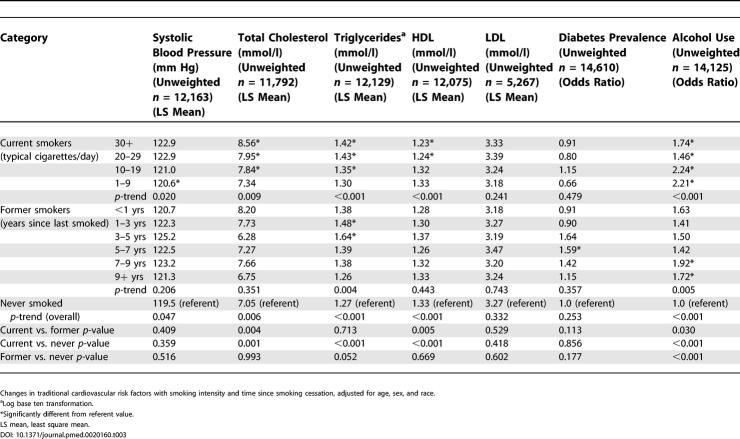
Physiologic Cardiovascular Measures by Smoking Status and Smoking Intensity

Changes in traditional cardiovascular risk factors with smoking intensity and time since smoking cessation, adjusted for age, sex, and race.

^a^Log base ten transformation.

*Significantly different from referent value.

LS mean, least square mean.

Lastly, the inflammatory markers were examined in fully adjusted models. C-reactive protein continued to show abatement of the acute phase response with reduced smoking intensity and increased time since cessation despite adjustment for covariates (Table 4). White blood cell count and albumin were associated with smoking intensity but not with time since cessation. All positive acute phase reactants showed an associated decline from current to former to never smokers in fully adjusted models (*p ≤* 0.01); albumin, likewise showed a significant increase (*p*-trend ≤ 0.01).

**Table 4:**
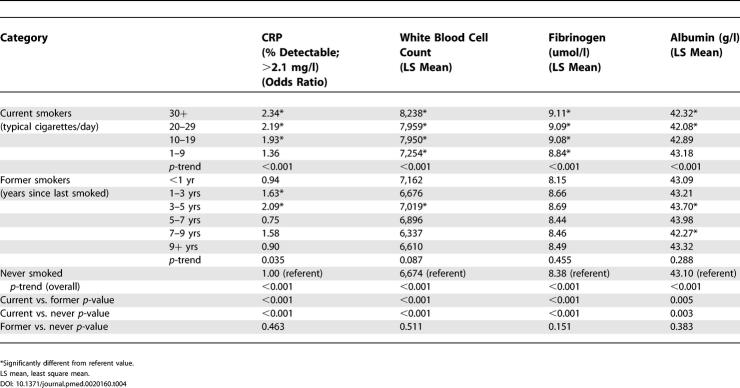
Levels of Inflammatory Markers Fully Adjusted for All Covariates

*Significantly different from referent value.

LS mean, least square mean.

Analyses using pack-years were also done (not shown); the results were unchanged using this measure. Additionally, data were reanalyzed excluding those individuals with prevalent cardiovascular disease (not shown); results were again unchanged and measures of association were even slightly stronger. Overall, we observed the following associations: (1) improvement in both inflammatory markers and traditional risk factors with decreased intensity of smoking, and (2) improvement in inflammatory markers with increased time since smoking cessation.

## Discussion

In this cross-sectional population-based study, we were able to demonstrate dose effects from smoking and temporal effects from cigarette smoking cessation. The NHANES III dataset allowed us to utilize both self-reported smoking and serum cotinine levels to minimize misclassification error. It also allowed us to control for a wide range of confounders and assess consistency across measures by analyzing four acute phase proteins and several traditional risk factors. By examining both traditional risk factors and inflammatory markers of cardiovascular risk, this study contributes to the literature by providing a more complete analysis with regard to the effects of smoking and smoking cessation.

It is known that after smokers give up smoking, their risk of mortality and future cardiac events declines [32,39], but there is little data quantifying the rate of risk reduction and when, or even whether, cardiovascular risk for former smokers reaches that of never smokers [12,40,41]. We found that the smoking-associated inflammatory response subsides within 5 y after smoking cessation. This suggests that the vascular effects are reversible and that cardiovascular risk subsides gradually with reduced exposure. The time to risk factor “correction” was longest with C-reactive protein; only one traditional risk factor—triglycerides—showed a similar pattern. Our findings are consistent with prior studies, suggesting that the full benefit of smoking cessation may be achieved gradually [39,42,43].

Our estimates of the time to risk factor “correction” are shorter than those for the decline in mortality risk and risk for specific cardiac events reported in some prospective studies [42,44], but on par with those in other prospective [45], case-control [46,47], and cross-sectional [48] studies. Discrepancies may be due to smoking recidivism in prospective studies, where former smokers recommence smoking and increase their risk, such that the reported time since cessation is longer than the true time, or due to a lag between changes in cardiovascular risk factors and regression of disease. Additionally, one would expect morbidity risk to subside first, since mortality is a more distant endpoint. Prior studies also did not control for known cardiovascular risk factors, did not use serum cotinine levels, did not examine the associated change in inflammatory markers post-cessation, or grouped participants into broad categories of current, former, and never smokers, increasing residual confounding while failing to explore the relative effects within each group [9,12,49]. Nevertheless, it remains unclear whether C-reactive protein's ongoing decline in former smokers simply indicates a prevalent underlying burden of atherosclerotic plaque, or “simmering” ongoing disease processes. The direct associations of C-reactive protein with mortality decrement seen in these other studies—and the dose–response seen in our own study—suggest the latter.

Three studies have systematically examined inflammatory markers following smoking: the MONICA Study (Monitoring Trends and Determinants in Cardiovascular Disease) [46], the Cardiovascular Health Study (CHS) [48], and the Northwick Park Heart Study [45]. The Cardiovascular Health Study found no association between inflammatory markers and smoking status itself, although C-reactive protein was strongly related to lifetime smoking exposure as measured by pack-years. Our results, while based on more complete modeling, support findings from the Northwick Park Heart Study and MONICA Study, both of which found that fibrinogen levels (adjusted only for age) reached normal levels within 5 y.

Our study supports the hypothesis that cardiovascular risk falls with inflammatory abatement, and that inflammatory markers are better indicators of such risk reduction. In this analysis, triglycerides showed the strongest dose-intensity and temporal trends of the traditional cardiovascular risk factors. This is both consistent with prior research and not surprising since the strongest association between C-reactive protein and lipid measures is for triglycerides. Despite the colinearity, the inflammatory markers studied appear to have a much clearer trend and longer lasting effect after smoking cessation than traditional risk factors. This would suggest their greater utility in being more accurate markers of disease, particularly given C-reactive protein's increasingly apparent role in the pathogenesis of atherosclerosis. Alternative explanations include (1) the presence of multiple independent causal pathways leading to cardiovascular disease and (2) traditional risk factors correlating more closely with mortality than morbidity or underlying pathophysiology. The relative value of novel inflammatory markers versus traditional risk factors remains of much debate [50,51].

Limitations of this study include measurement error from self-reporting, residual confounding from use of indicator variables, smoking recidivism, lack of newer measures such as interleukin-6 and high-sensitivity C-reactive protein [52], and lack of data on secondhand smoke. Recent studies have suggested that toxin exposures from secondhand smoke may impact the biomarkers used in this study [53,54]. We attempted to adjust for bias in self-reporting and secondhand exposure by using serum cotinine levels, but residual confounding may exist. It has also been suggested that circulating C-reactive protein levels may not reflect all relevant inflammatory effectors, owing to post-transcriptional regulation of C-reactive protein [22]. We chose C-reactive protein, however, for its clear role in the inflammatory response and for its increasing clinical relevance. Lastly, it is noteworthy that we are not using a linear scale, but measures of both intensity and duration. Time since cessation and cigarettes per day are distinctly different measures, and a discontinuity arises at cessation.

Further research should explore the acute phase response in the months following smoking cessation [53,55–57]. Time intervals of less than a year since cessation have not been assessed adequately in other studies nor in this study because of sample size limitations of the NHANES III data. Additionally, if cardiovascular risk subsides gradually upon smoking cessation, do vessel wall damage and pro-aggregatory cascades all completely reverse course upon cessation? Future research should continue to explore the relationship between the inflammatory cascade and alterations in the vessel wall [7,58].

This and other similar studies suggest that smoking cessation should play a larger role in public policy. Linked to poverty, decreased productivity, and premature death, tobacco remains the second major cause of preventable death in the world and fourth most common risk factor for disease worldwide [59]. While tobacco is clearly a worldwide concern, states and localities are left responsible for addressing tobacco use and smoking in a comprehensive manner. Our results suggest that policy-makers faced with escalating health-care costs should look to smoking cessation as an opportunity to achieve both long- and short*-*term health-care cost savings through cardiovascular risk reduction [60]. If cardiovascular benefits are, in fact, readily attainable, there exists an opportunity for short-term gain from smoking cessation. This may be sufficient to induce policy change. Larger scale action is also needed. Arguments persist in the developing world regarding the forced opening of tobacco markets as a result of the Global Agreements on Tariffs and Trade. Guidance may be forthcoming, however, from the World Health Organization Framework Convention on Tobacco Control, which began operation in February 2005 as an international treaty aimed at controlling tobacco packaging, marketing, and use [61]. Over 60 nation-states are currently parties to the Framework Convention on Tobacco Control, including many European nations and Japan, although the United States has yet to approve this treaty. Opportunities for tobacco control remain available, and it is becoming increasingly clear that smoking cessation models deserve high priority both in research and in any preventive health care system.

**Patient Summary**

**Background**

Smoking is associated with an increased chance of getting cardiovascular abnormalities, such as atherosclerosis, which can lead to heart attacks and other illnesses. One of the ways in which smoking causes damage is by causing inflammation; this inflammation can be measured by looking at the levels of various substances (such as C-reactive protein, white blood cells, albumin, and fibrinogen) in the blood. Stopping smoking decreases the chances of developing cardiovascular disease, and this decreased risk may be related to the levels of the inflammatory markers in the blood.

**What Did the Researchers Do?**

They compared the levels of the markers of inflammation measured in the blood of around 15,000 people who had taken part in a National lifestyle study in the United States with information about whether these people had ever smoked and whether they smoked currently.

The researchers found that the markers of inflammation were lowest in those people who had never smoked, and decreased as the time since stopping smoking increased. It took five years after stopping smoking for the inflammatory makers to return to normal.

**What Do These Findings Mean?**

Smoking is the second most important cause of death worldwide, and one of the main ways it causes death is through cardiovascular disease. This work confirms that the risk of cardiovascular disease does decrease after stopping smoking, and is marked by changes in the levels of inflammatory markers. Encouraging people to give up smoking is very important to reduce the amount of cardiovascular disease caused by smoking, but the markers of inflammation, and hence the risk of getting cardiovascular disease, may take several years to return to normal.

**Where Can I Find More Information?**

MedlinePlus has a Web page on smoking and other related health issues: http://www.nlm.nih.gov/medlineplus/smoking.html

The World Health Organization has a section on tobacco control: http://www.who.int/tobacco/en/

## Abbreviations

HDL: high-density lipoprotein
LDL: low-density lipoprotein
NHANES III: Third National Health and Nutrition Examination Survey
